# Ultrafast MR imaging findings of 2 different subtypes in a male patient with bilateral breast cancer

**DOI:** 10.1016/j.radcr.2023.12.043

**Published:** 2024-01-15

**Authors:** Kyle Kleiman, Ceren Yalniz, Stefanie Woodard

**Affiliations:** a Edward Via College of Osteopathic Medicine, Carolinas Campus, 350 Howard St, Spartanburg, SC 29303, USA; b Department of Radiology, The University of Alabama at Birmingham, 619 19th Street South, Birmingham, AL 35249, USA

**Keywords:** Ultrafast, Breast MRI, Micropapillary, Male breast cancer, Bilateral breast cancer

## Abstract

Bilateral breast cancer in males is an exceedingly rare diagnosis. In this case report, we will discuss the ultrafast sequence findings of a bilateral male breast cancer with different subtypes on his staging dynamic contrast enhanced (DCE) MRI with ultrafast technique. A 94-year-old male presented with bilateral palpable complaints in his breasts. Diagnostic mammography and ultrasound images demonstrated bilateral irregular masses with nipple retraction. Biopsies were performed and the histopathologic examination revealed invasive breast carcinoma of no special type in 1 breast and invasive micropapillary carcinoma in the other breast. Staging MRI with ultrafast sequence showed significant enhancement differences between 2 different subtypes, correlating with the different levels of tumor aggressiveness. Different ultrafast metrics, such as time-to-enhancement and maximum slope, may help to differentiate between several subtypes of breast cancer and serve as prognostic indicators. This case report discusses the application of ultrafast sequence in predicting breast cancer subtypes in a male patient with bilateral disease.

## Introduction

Male breast cancer is rare, accounting for less than 1% of breast cancer cases. However, the incidence is increasing potentially due to obesity, increased longevity, and BRCA2 mutation [1]. A bilateral, synchronous presentation of breast cancer in male patients is much rarer with an incidence of approximately 1.5%-2.0% [2] and it is commonly of the same histologic type. In a comparative study by Hungness et al. [3] 43% of patients with bilateral breast cancer were found to have a different histologic type of cancer in each breast.

Invasive breast carcinoma of no special type is the most common type of breast cancer [4]. Invasive micropapillary carcinoma is a rare variant of invasive ductal carcinoma with a higher rate of recurrence, more common lymph node metastasis and vascular invasion [5].

Breast MRI has the highest sensitivity among current breast cancer screening modalities, including mammography (96.5% vs 86.9%) [6,7]. This is particularly evident in scenarios such as imaging in dense breasts. In patients with dense breasts, mammographic sensitivity can drop to 50% while sensitivity of MRI is not limited by breast tissue density [8]. Full-protocol dynamic contrast enhanced (DCE) MRI and abbreviated MRI are the current protocols in practice. Ultrafast MRI is a distinct imaging sequence and is a new technique developed to capture initial contrast material uptake into breast lesions. It does not change the overall acquisition time since it takes place during the initial pre-scan time-delay of dynamic imaging for a full-protocol breast MRI. According to early results from reader studies, the sensitivity of ultrafast MRI and full-protocol MRI are quite similar (92.4% vs. 93.0%), while the specificity of ultrafast MRI was found to be greater than full-protocol MRI (82% vs 76%) due to the high temporal resolution [8]. However, specificity in mammography tends to be higher (88.9%) than in either MRI protocol [7]. Overall, ultrafast MRI may have potential to give more information about the prognostic factors and the malignant potential of breast cancer.

## Case presentation

A 94-year-old male presented to the clinic with bilateral palpable breast masses. He had a personal history of cancer, specifically bladder cancer, melanoma, and squamous cell carcinoma. He tested negative for BRCA mutation. A clinical physical exam was performed and bilateral breast masses and left axillary lymph nodes were palpated. The nipples were inverted bilaterally, as well as the left nipple almost being fully eroded. No abnormal infraclavicular, supraclavicular, or cervical region lymph nodes were documented.

**Fig. 1:**
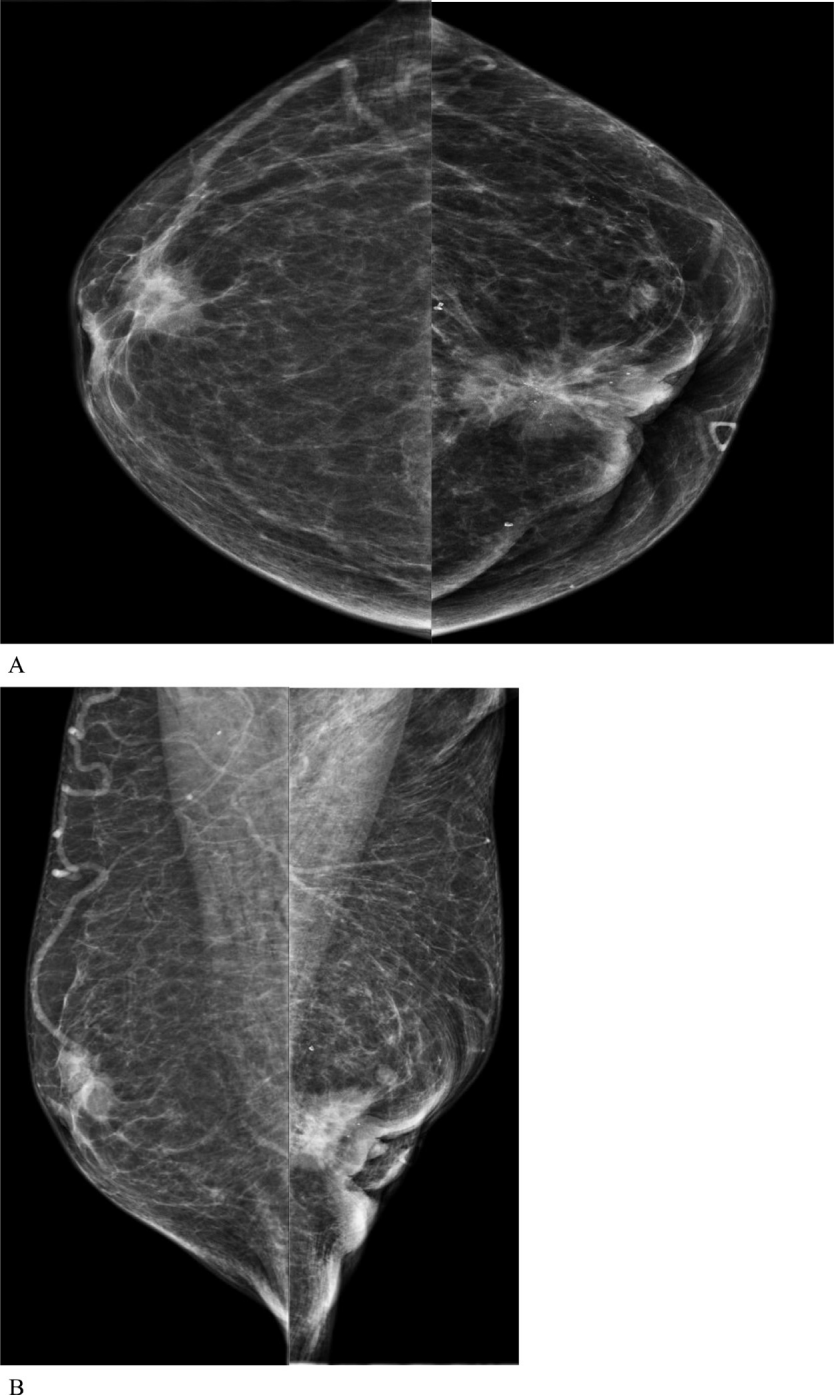
Diagnostic full-field digital 2-D mammography (A) craniocaudal views and (B) mediolateral oblique views demonstrated a right breast upper outer quadrant, anterior depth irregular mass with associated nipple retraction and a left upper outer, anterior depth irregular mass with associated skin retraction, nipple retraction and microcalcifications.

**Fig. 2:**
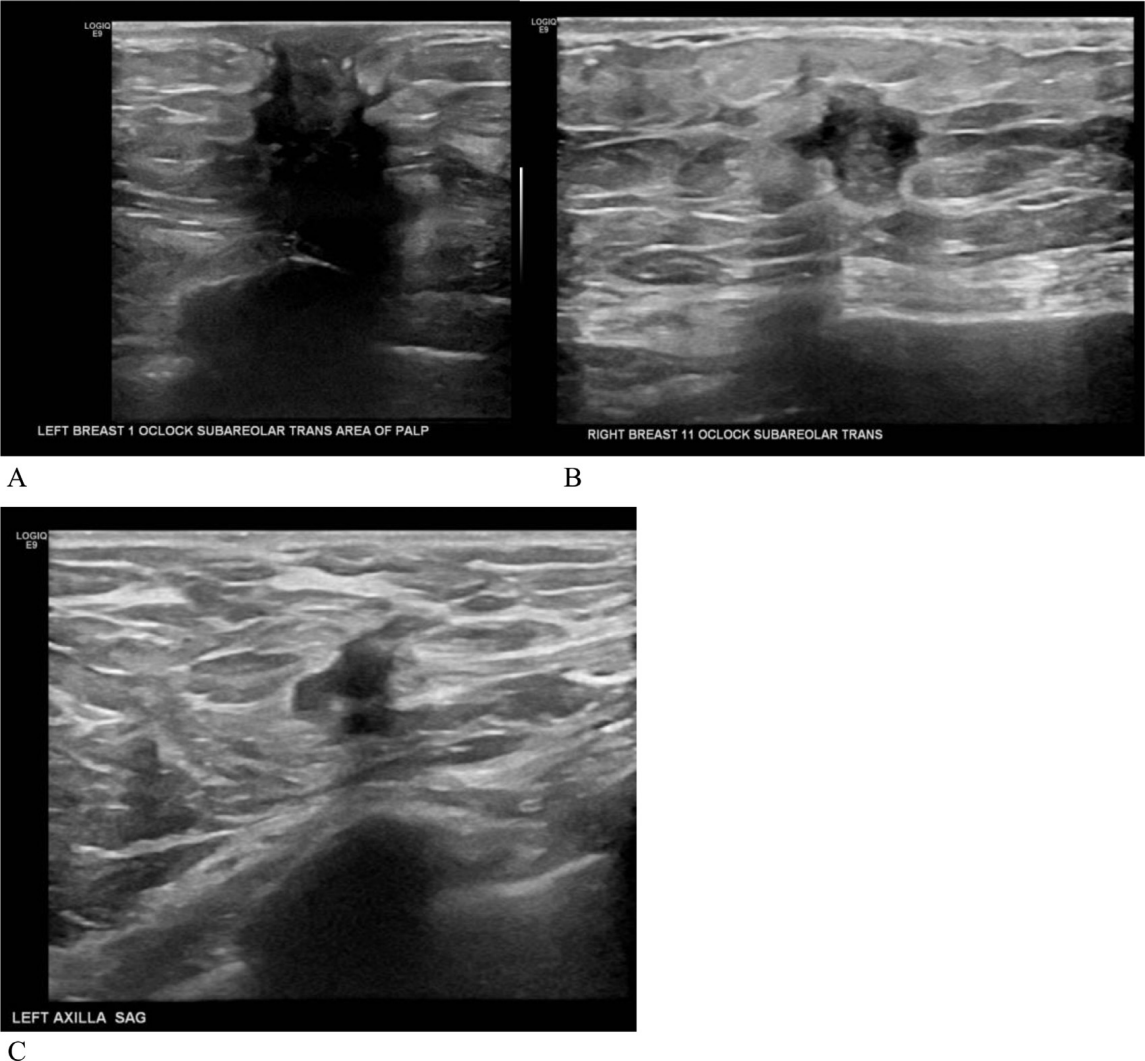
Grayscale targeted ultrasound images of the left breast (A) and right breast (B) and the left axilla (C) demonstrated bilateral, irregular masses and abnormal left axillary lymph node.

The patient had a bilateral diagnostic mammography and ultrasound to evaluate the bilateral breast masses. The mammography demonstrated a right upper outer quadrant, anterior depth irregular mass with associated nipple retraction and a left upper outer quadrant irregular mass with associated skin retraction, nipple retraction, and microcalcifications (Fig. 1). Ultrasound revealed an irregular mass, measuring 13×10×16 mm, at 11:00 position in the right breast and another irregular mass, measuring 28 × 32 × 23 mm, at 1:00 position in the left breast (Fig. 2). The left breast mass involved the nipple-areolar complex as well as the pectoralis muscle. There was also a morphologically abnormal level 1 lymph node in the left axilla, which demonstrated eccentric cortical thickening with indistinct margins, concerning for extracapsular extension. Bilateral ultrasound-guided core biopsy was performed.

Histopathology of the left 1:00 breast mass revealed a Nottingham Grade 2 invasive breast carcinoma of no special type. The right 11:00 breast mass was consistent with a Nottingham Grade 2 invasive ductal carcinoma with focal micropapillary features.

Both cancers were found to be estrogen (ER) and progesterone (PR) positive, HER2 negative**.** Subsequent CT examination of the chest, abdomen, and pelvis showed extensive metastatic disease, likely from breast origin. Standard of care was extrapolated from female breast cancer due to the rarity of male breast cancer and the patient was started on combination therapy including testicular hormone separation with leuprolide, an aromatase inhibitor, palbociclib, and letrozole.

**Fig. 3:**
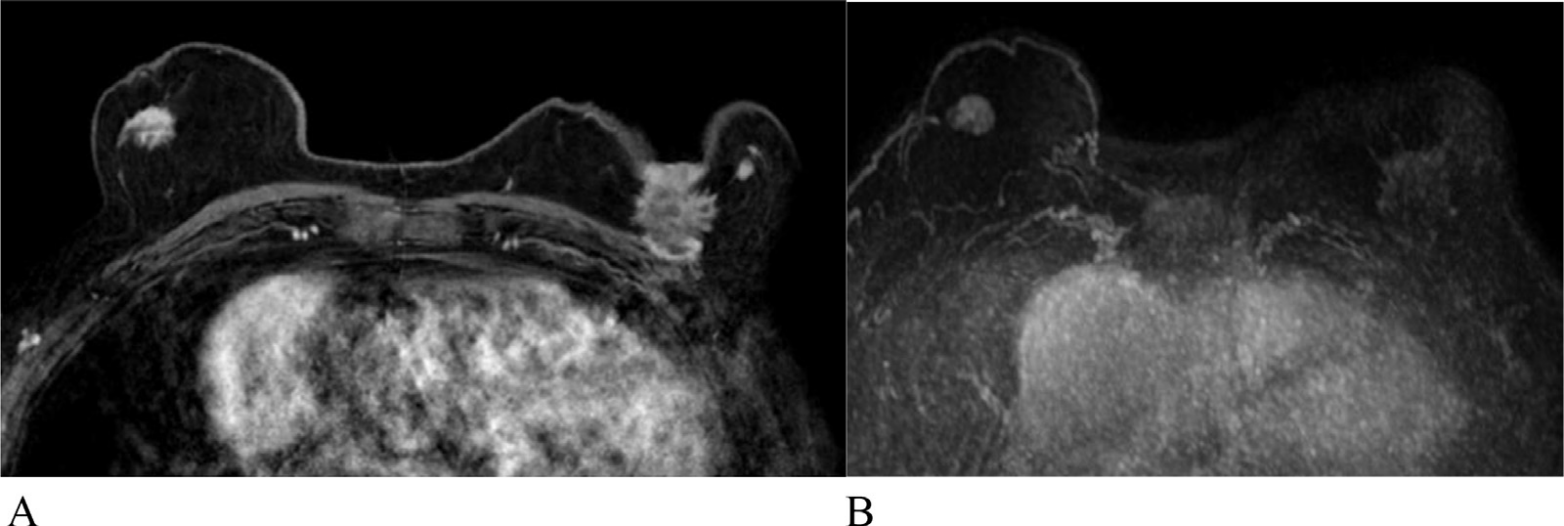
Full-protocol dynamic contrast enhanced (DCE) breast MRI with ultrafast sequence. (A) Subtraction images demonstrate an oval enhancing mass with irregular margins in the right breast, consistent with invasive ductal carcinoma with micropapillary features and an irregular mass with spiculated margins in the left breast with nipple and chest wall invasion, consistent with invasive breast carcinoma of no special type and (B) Both lesions show rapid contrast uptake on ultrafast sequence; however, the enhancement is more avid for the right breast malignancy.

Full-protocol dynamic contrast enhanced (DCE) breast MRI with ultrafast sequence was performed for staging of the bilateral disease (Fig. 3). The MRI demonstrated an oval enhancing mass with irregular margins in the right breast and an irregular mass with spiculated margins in the left breast with nipple and chest wall invasion.

## Discussion

As mentioned earlier, male breast cancer is exceedingly rare and bilateral presentation is even more rare. Even though, there are no guidelines for breast MRI indications in male patients, MRI can be extremely helpful to evaluate the disease extent for staging purposes [9].

Invasive breast carcinoma of no special type is the most common type of breast cancer [4]. Invasive micropapillary carcinoma is a subtype of invasive ductal carcinoma with an increased incidence of lymphovascular invasion and clinically positive axillary lymph nodes. Mammographic appearance of invasive micropapillary carcinoma is often nonspecific and underestimates the true disease burden in this cancer subtype. A true estimation of tumor size and the disease extent is important for proper management, as a more radical surgical approach is often warranted. MRI is considered the best imaging modality for this subtype and it generally presents as an irregular mass with spiculated margins and rapid enhancement and washout on delayed phase images [10].

Ultrafast, which is a novel MRI technique, can help to differentiate malignant breast lesions from benign lesions of the breast. There are new diagnostic parameters for ultrafast technique, such as time-to-enhancement (TTE) and maximum slope (MS). TTE, the time for contrast to travel from the aorta to the breast lesion, is significantly shorter in malignant lesions compared to benign lesions or breast tissue. A shorter TTE is also associated with a higher grade of cancer [11]. Malignant tumors are described to stand out like a lightbulb on ultrafast images. This is because the generally more hyper vascular malignant masses take up higher amounts of contrast [12]. The other parameter, MS has been reported to be associated with axillary lymph node metastasis and an invasive component [13]. These different metrics, used by ultrafast technique are helpful to not only differentiate benign and malignant lesions, but also to differentiate different subtypes and to serve as prognostic markers. They may also be of value in situations such as monitoring treatment response to neoadjuvant therapy [14].

Our case demonstrates different presentations of breast cancer subtypes on ultrafast sequence, in a male patient with bilateral breast cancer. In this patient, micropapillary cancer, which is a more aggressive subtype, shows a shorter TTE, a higher MS, and more robust enhancement in comparison to invasive breast carcinoma of no special type. This correlates with a more aggressive nature of this subtype and may warrant more aggressive treatment.

## Conclusion

We report a rare occurrence of bilateral breast cancer in a male patient with different histology. The ultrafast sequence findings were highlighted to show the differences between a more aggressive micropapillary subtype of the invasive ductal carcinoma and a relatively less aggressive invasive breast carcinoma of no special type. In conclusion, ultrafast technique is an important tool for breast cancer evaluation and it can predict tumor aggressiveness.

## Patient consent

Written informed consent was obtained from the patient for use in this case report. No identifiable protected health information was utilized.
